# Antibacterial effects of epigallocatechin gallate against calf diarrhea-causing *Escherichia coli*: *in vitro* and *in vivo* investigations

**DOI:** 10.3389/fvets.2026.1752084

**Published:** 2026-04-09

**Authors:** Yu Ren, Lingkang Liu, Wenya Zheng, Ben Liu, Zerui Zhao, Xunhao Zhu, Lisen He, Songlin Ding, Wei Hu, Lucheng Zheng, Qingcan Fan, Hailong Hong

**Affiliations:** 1 College of Life Science and Resources and Environment, Yichun University, Yichun, Jiangxi, China; 2 Yichun University Research Center for Traditional Chinese Veterinary Medicine and Animal Embryo Engineering Technology, Yichun, Jiangxi, China

**Keywords:** antibacterial effect, antibiotic alternative, calf diarrhea, epigallocatechin gallate (EGCG), *Escherichia coli*, tight junction protein

## Abstract

**Introduction:**

Calf diarrhea caused by *Escherichia coli* poses severe economic burdens to the cattle industry, and antibiotic overuse has triggered drug resistance and residue risks. This study screened 10 plant extracts for antibacterial activity against a clinically isolated calf diarrhea-causing *E. coli* strain *in vitro*, and investigated the protective effects of epigallocatechin gallate (EGCG)—the most effective extract—on *E. coli*-infected mice.

**Methods:**

Antimicrobial susceptibility was assessed by disk diffusion, minimum inhibitory concentration (MIC), and minimum bactericidal concentration (MBC) assays. *In vivo*, mice were intraperitoneally challenged with *E. coli* and treated with 50 mg/kg or 100 mg/kg EGCG. Bacterial loads in liver and small intestine were quantified, intestinal pathology was evaluated by HE staining, and expression of tight junction proteins (ZO-1, Occludin, Claudin-1) was analyzed by immunohistochemistry and Western blotting.

**Results:**

EGCG exhibited the largest inhibition zone (19.7 ± 0.3 mm) against the pathogen, with an MIC of 3.125 mg/mL and MBC of 50 mg/mL. *In vivo*, both 50 mg/kg and 100 mg/kg EGCG significantly reduced bacterial loads in the liver and small intestine of infected mice, alleviated small intestinal pathological damage, and the 100 mg/kg EGCG group showed markedly upregulated expression of ZO-1, Occludin, and Claudin-1 in the small intestine (*p <* 0.05).

**Discussion:**

Our findings confirm that EGCG exerts potent *in vitro* antibacterial effects against calf diarrhea-causing *E. coli* and protects against *E. coli*-induced small intestinal injury in mice, highlighting its potential as a natural antibiotic alternative for controlling colibacillary diarrhea in calves.

## 1 Introduction

Diarrhea in calves is one of the most significant issues in cattle farming. It is seasonal, occurring frequently in winter, spring, and rainy seasons, with relatively high incidence and mortality rates. The clinical symptoms of this disease are typical: initially, it presents as the excretion of light brown, foul-smelling feces, and in the later stages, it develops into bloody watery stools, accompanied by severe abdominal pain, dehydration, and other symptoms. Persistent diarrhea not only leads to stunted growth and development of calves, prolonged feeding cycles, but also can cause death in severe cases, imposing a heavy economic burden on the cattle industry (1). A variety of pathogens can cause calf diarrhea, among which *Escherichia coli* (*E. coli*) is the most important pathogenic bacterium (2, 3). Recent surveys have confirmed the high prevalence of pathogenic *E. coli* in livestock, including cattle, sheep, and goats, with diverse serogroups and antimicrobial resistance profiles (4–6). Antibiotics are currently the most effective measure for treating such diseases. However, due to the overuse of antibiotics, problems such as drug resistance and drug residues have become increasingly serious, often resulting in reduced or ineffective treatment outcomes (36). They also trigger a series of issues including food safety and public health safety (7), which is contrary to China’s policies of green and coordinated development and harmonious coexistence between humans and nature. Therefore, the screening and development of safe, reliable, and low-toxic antibiotic alternative drugs have become a necessity. Natural plant extracts can be used as antibacterial agents and have advantages over traditional synthetic antibiotics, such as low toxicity, few side effects, and low likelihood of inducing drug resistance (8). They are ideal alternatives to antibiotics, holding the promise of opening up new pathways for the prevention and control of calf diarrhea and promoting the green and healthy development of the cattle industry.

The intestine is the main target organ of *E. coli* (9), and the integrity of its barrier function is crucial for resisting bacterial infections. The intestinal barrier is composed of various tight junction proteins, among which Occludin, Claudin-1, and ZO-1 are key protein components of tight junctions. Occludin is a transmembrane protein that interacts with ZO-1 and other proteins to strengthen cell connections, prevent the invasion of harmful substances, and maintain the integrity of the intestinal barrier; its reduction will increase intestinal permeability (34). The Claudin family acts as a “gate” of the intestinal barrier, regulating substance transport and maintaining the barrier. ZO-1, as a bridging protein, connects other proteins to the cytoskeleton, ensuring the physical integrity of the barrier, guaranteeing the stability of tight junctions, and participating in the regulation of intestinal mucosal repair as well as cell proliferation and apoptosis (10)

In this experiment, 10 plant extracts such as epigallocatechin gallate (EGCG), Shikonin, and Chlorogenic acid were selected for *in vitro* antibacterial experiments against a clinically isolated calf diarrhea-derived *E. coli* strain to screen out plant extracts with better antibacterial effects. To our knowledge, this is the first study to evaluate EGCG against a calf-derived *E. coli* isolate and to comprehensively assess its protective effects on intestinal barrier integrity using an integrative *in vitro*, *in vivo*, and molecular approach. On this basis, methods such as HE staining, immunohistochemistry, and Western blotting were used to investigate the effects of EGCG on the bacterial load, pathological changes of the small intestine, and the expression of tight junction proteins Occludin, ZO-1, and Claudin-1 in mice infected with *E. coli*. The results of this study can not only provide an important theoretical basis for the prevention and control strategies of calf colibacillary diarrhea but also lay a foundation for the development of new natural plant-derived antibacterial drugs, helping to promote the green and healthy development of animal husbandry

## 2 Materials and methods

### 2.1 Plant extracts and test strains

A total of 10 samples were collected, including Epigallocatechin gallate (EGCG), Baicalein, Shikonin, Resveratrol, Naringenin, Curcumin, Fisetin, Kaempferol, Quercetin, and Chlorogenic acid. Shikonin was purchased from Shanghai Yuanye Bio-Technology Co., Ltd., Chlorogenic acid was obtained from Beijing Solarbio Science & Technology Co., Ltd., and the remaining 8 plant extracts were purchased from Shanghai Aladdin Biochemical Technology Co., Ltd. The test strain was a pathogenic *Escherichia coli* isolate obtained from a calf with clinical diarrhea at a local farm in Yichun, Jiangxi Province, China. The isolate was identified by Gram staining, biochemical tests (VITEK 2), and 16S rRNA gene sequencing. The strain was provided by the Research Center for Chinese Veterinary Medicine and Animal Embryo Engineering Technology, Yichun University. Genotyping of virulence genes (e.g., estA, elt, stx) and antimicrobial resistance profiling were not performed in this study, which is acknowledged as a limitation.

### 2.2 Main reagents and instruments

Antibodies such as Occludin, Claudin-1, ZO-1, and Goat Anti-mice IgG secondary antibody were purchased from Abcam Trading (Shanghai) Co., Ltd. The mice SP kit, diaminobenzidine (DAB) chromogenic kit, BCA protein assay kit, SDS-PAGE gel preparation kit, protein Marker, *β*-actin antibody, and chemiluminescence solution were all purchased from Wuhan Boster Biological Engineering Co., Ltd. The high-efficiency RIPA tissue/cell lysis buffer kit, hematoxylin–eosin (HE) staining reagents, etc., were purchased from Wuhan Servicebio Technology Co., Ltd. MH nutrient agar, MH broth, magnetic bead bacterial preservation tubes, and brain heart infusion broth were purchased from Qingdao Haibo Biotechnology Co., Ltd. Turbidity tubes were purchased from Zhuhai Baso Biotechnology Co., Ltd. DMSO was purchased from Sigma-Aldrich (Shanghai). Tris-buffered saline (TBS) and TBS containing Tween-20 (TBST) were purchased from Beijing Solarbio Science & Technology Co., Ltd. Phosphate buffer saline (PBS) was purchased from Wuhan Puno Sai Life Science Co., Ltd. Paraformaldehyde was purchased from Tianjin Damao Chemical Reagent Factory. Anhydrous ethanol was purchased from Sinopharm Chemical Reagent Co., Ltd. Electronic balances, ultra-low temperature freezers, portable pressure steam sterilizers, biochemical incubators, constant temperature shaking incubators, clean benches, etc., were provided by the Research Center for Chinese Veterinary Medicine and Animal Embryo Engineering Technology, Yichun University.

### 2.3 Preparation of culture media and antibacterial agents

Culture media were prepared according to standard microbiological protocols (39). Brain heart infusion broth, Mueller-Hinton (MH) broth, and MH agar were prepared by dissolving the appropriate amounts of commercial dehydrated powders in distilled water, followed by sterilization by autoclaving at 121 °C for 15 min. After sterilization, liquid media were aliquoted into sterile tubes, and agar media were poured into sterile Petri dishes. All media were incubated at 37 °C for 24 h to verify sterility and then stored at 4 °C until use.

Preparation of antibacterial agents: 10 plant extracts including epigallocatechin gallate, baicalein, and shikonin were dissolved in DMSO to prepare solutions with concentrations of 100 mg/mL, 50 mg/mL, 25 mg/mL, 12.5 mg/mL, 6.25 mg/mL, 3.125 mg/mL, 1.5625 mg/mL, 0.78125 mg/mL, 0.390625 mg/mL, and 0.1953125 mg/mL. The solutions were sterilized by filtration through a 0.22 μm microporous membrane. The treated antimicrobial susceptibility discs were immersed in the above-mentioned 10 extracts and stored in a 4 °C refrigerator for 24 h.

### 2.4 Activation of test strains and preparation of bacterial suspensions

The cryopreserved magnetic bead tubes were taken out from the −80 °C refrigerator. After shaking, 1–2 magnetic beads were picked and placed into a centrifuge tube containing 5 mL of brain heart infusion broth. The tube was incubated in a 37 °C constant temperature shaking incubator at 150 rpm until obvious turbidity appeared. Then 100 μL of the bacterial solution was transferred to freshly prepared nutrient broth, and rejuvenation was performed 3 times under the same conditions. The last shaking culture was not exceeding 7 h to obtain freshly activated bacterial solution. The activated bacterial solution was streaked and inoculated on nutrient agar plates, which were then cultured in a 37 °C biochemical incubator until distinct colonies grew. Bacterial suspension was prepared and compared with McFarland turbidity tubes to adjust the bacterial suspension to 0.5 McFarland standard.

### 2.5 Antimicrobial susceptibility test

A sterile cotton swab was dipped into the bacterial suspension and evenly spread over the entire nutrient agar plate. The plate was inverted for 2–3 min to allow complete absorption of the bacterial suspension by the nutrient agar. Using sterile tweezers, the drug-sensitive discs soaked in the medicinal solutions were attached to the plate, with 5 discs per plate and a distance of more than 30 mm between each other. The plate was then inverted and incubated in a 37 °C biochemical incubator for 10 h. The diameter of each inhibition zone was measured with a vernier caliper. Three independent experiments were performed for each extract, and results are expressed as mean ± standard deviation (SD). A solvent control was also performed simultaneously.

### 2.6 Determination of minimum inhibitory concentrations (MICs) and minimum bactericidal concentrations (MBCs)

Ten plant extracts including epigallocatechin gallate were subjected to 2-fold serial dilution to prepare 8 concentrations: 100, 50, 25, 12.5, 6.25, 3.125, 1.5625, and 0.78125 mg/mL. After preparation, the solutions were gently shaken to mix well, and the culture results were observed. Turbidity in the wells indicated bacterial growth, while a transparent state indicated no bacterial growth. The lowest concentration of the extract that resulted in a transparent state was defined as the minimum inhibitory concentrations (MICs) of the extract against the strain. A 50 μL aliquot was taken from each well with no bacterial growth and spread on nutrient agar. The lowest concentration of the extract at which the number of growing colonies was <5–10 was determined as the minimum bactericidal concentrations (MBCs) of the extract. All assays were performed in triplicate, and MIC/MBC values are reported as mean ± SD.

### 2.7 Determination of bacterial load in mice organs

Twenty-four SPF-grade Kunming mice (6 per group, sample size based on pilot studies and resource availability) were randomly divided into 4 groups: control group, model group, 50 mg/kg EGCG treatment group, and 100 mg/kg EGCG treatment group, with intragastric administration at a dose of 0.02 mL/g. The EGCG doses were selected based on previous studies (11, 12) and preliminary dose-range experiments (data not shown). The control group was given 0.9% normal saline, while the model group and treatment groups were injected with 1 × 10^8^ colony-forming unit (CFU)/mL bacterial solution suspension. Two hours later, the treatment groups were administered EGCG at the corresponding concentrations, once every 12 h for 4 consecutive times. Forty-eight hours after drug withdrawal, the mice were sacrificed by cervical dislocation. The duodenum, jejunum, and ileum tissues were collected, added with sterile normal saline for homogenization, and the tissue fluid was subjected to serial dilution. A 100 μL aliquot of the diluted solution at the lowest concentration was dropped onto MacConkey agar plates and spread evenly. After the bacterial solution was absorbed, the plates were inverted and incubated in a 37 °C constant-temperature incubator for 24 h. The number of colonies was counted and the bacterial load in the tissues was calculated.

### 2.8 Methods for paraffin sectioning and HE staining of mice small intestine

Tissues were subjected to dehydration, clearing, wax infiltration, and embedding to prepare paraffin sections. The sections were baked, dewaxed, rehydrated, stained with hematoxylin, differentiated and blued, stained with eosin, dehydrated and cleared, followed by mounting. The morphology and pathological changes of tissue cells were observed under a microscope.

### 2.9 Immunohistochemical (IHC) staining

After pre-warming the paraffin sections, they were dewaxed in xylene and rehydrated through a descending ethanol series. Blocking and sealing were performed in accordance with the instructions of the mice SP kit. The primary antibody working solution was added, followed by color development using the DAB kit, counterstaining with hematoxylin, differentiation with hydrochloric acid ethanol, dehydration through an ascending gradient ethanol series, clearing in xylene, and mounting. The staining results were evaluated under a microscope. A yellow or light yellow color developed by DAB indicated a positive result, while no color development indicated a negative result.

### 2.10 Western blotting (WB)

After extracting total proteins from the tissues, the protein concentration was determined using the BCA method. A 5 × SDS-PAGE protein loading buffer was added to the protein extract in proportion to prepare the protein loading solution. After loading samples in sequence, SDS-PAGE gel electrophoresis was performed. The membrane was cut to an appropriate size, and then a polyvinylidene fluoride (PVDF) membrane was used for wet transfer. The PVDF membrane was incubated with primary antibodies (ZO-1, Occludin, and Claudin-1 were diluted at 1:1000) and the internal reference protein *β*-actin (diluted at 1:2500) at 4 °C, followed by incubation with the secondary antibody Goat Anti-mice IgG (diluted at 1:5000). Finally, the PVDF membrane was imaged according to the instructions of the Super ECL Plus Ultra-sensitive Chemiluminescence Detection Kit, and photographs were taken using an AI600 chemiluminescence imaging system.

### 2.11 Statistical analyses

Statistical analyses were performed using SPSS statistical software. Data are presented as mean ± SD. Comparisons among multiple groups were analyzed by one-way analysis of variance (ANOVA) followed by Tukey’s post-hoc test for multiple comparisons. A value of *p < 0.05* was considered to indicate statistically significant difference, while *p > 0.05* indicated no significant difference.

## 3 Results

### 3.1 Antimicrobial susceptibility test

**Figure 1:**
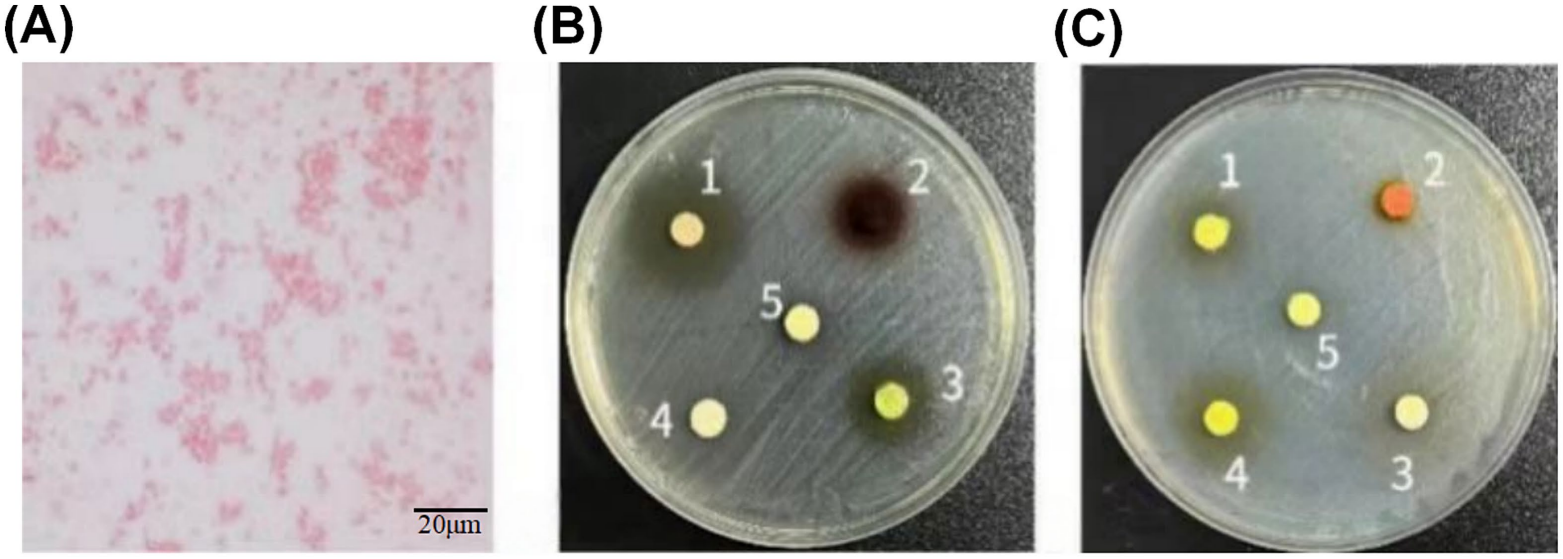
Gram staining and antimicrobial susceptibility test results of *Escherichia coli* causing calf diarrhea. **(A)** Gram staining microscopy of *E. coli* (oil immersion lens). The bacteria appear as Gram-negative rods, distributed singly or in chains. Scale bar = 20 μm. **(B)** Zone of inhibition of *E. coli* by 5 plant extracts detected via Kirby-Bauer (K-B) disk diffusion assay. The numbered extracts correspond to: 1. Epigallocatechin gallate, 2. Shikonin, 3. Baicalin, 4. Kaempferol, 5. Chlorogenic acid. **(C)** The sizes of inhibition zones produced by another 5 plant extracts. The numbered extracts correspond to: 1. Resveratrol, 2. Curcumin, 3. Fisetin, 4. Naringenin, 5. Quercetin. Data are presented as mean ± SD from three independent experiments.

After Gram staining, the test strains were observed under an oil immersion microscope to be rod-shaped, distributed singly or in chains, as shown in Figure 1A. The results of the K-B disk diffusion antimicrobial susceptibility test are presented in Table 1 and Figures 1B,C. The *Escherichia coli* causing calf diarrhea exhibited certain differences in susceptibility to these 10 plant extracts. It showed high sensitivity to epigallocatechin gallate, baicalein, shikonin, resveratrol, and naringenin, with inhibition zone diameters all greater than 10 mm, and moderate sensitivity to the remaining 5 plant extracts. Data from triplicate experiments are presented as mean ± SD in Table 1.

**Table 1:** Susceptibility of *Escherichia coli* strains causing calf diarrhea to 10 plant extracts.

**Plant extracts**	**Inhibition zone diameter (mm)**	**Antibacterial intensity**
Epigallocatechin gallate	19.7 ± 0.3	++
Baicalein	12.5 ± 0.4	++
Shikonin	11.1 ± 0.2	++
Resveratrol	10.5 ± 0.3	++
Naringenin	10 ± 0.2	++
Curcumin	6.3 ± 0.1	+
Fisetin	6.2 ± 0.1	+
Kaempferol	6.2 ± 0.1	+
Quercetin	6.2 ± 0.1	+
Chlorogenic acid	6.1 ± 0.1	+

R ≥ 9 mm is highly sensitive (++), 5 mm ≤ R < 9 mm is moderately sensitive (+), and R < 5 mm is insensitive.

### 3.2 Minimum inhibitory concentrations (MICs) and minimum bactericidal concentrations (MBCs) values

The results are shown in Table 2 as mean ± SD from three independent experiments. The 10 plant extracts exhibited varying inhibitory activities against *Escherichia coli* causing calf diarrhea. Epigallocatechin gallate, shikonin, baicalein, naringenin, and resveratrol showed the strongest inhibitory activity, with a minimum inhibitory concentration (MIC) of 3.125 mg/mL. This was followed by fisetin, kaempferol, quercetin, curcumin, and chlorogenic acid, which had an MIC of 6.25 mg/mL. Among the 10 plant extracts, baicalein displayed the strongest bactericidal activity with a minimum bactericidal concentration (MBC) of 25 mg/mL, followed by epigallocatechin gallate, shikonin, naringenin, and chlorogenic acid, which all had an MBC of 50 mg/mL. The remaining plant extracts showed relatively weak bactericidal activity.

**Table 2:** MICs and MBCs values of 10 plant extracts against *Escherichia coli* causing calf diarrhea.

Plant extracts	MICs(mg•mL^−1^)	MBCs(mg•mL^−1^)
Epigallocatechin gallate	3.125 ± 0.0	50 ± 0.0
Baicalein	3.125 ± 0.0	50 ± 0.0
Shikonin	3.125 ± 0.0	25 ± 0.0
Resveratrol	3.125 ± 0.0	50 ± 0.0
Naringenin	3.125 ± 0.0	100 ± 0.0
Curcumin	6.25 ± 0.0	100 ± 0.0
Fisetin	6.25 ± 0.0	100 ± 0.0
Kaempferol	6.25 ± 0.0	100 ± 0.0
Quercetin	6.25 ± 0.0	100 ± 0.0
Chlorogenic acid	6.25 ± 0.0	50 ± 0.0

MICs, minimum inhibitory concentrations; MBCs, minimum bactericidal concentrations. mg•mL^−1^: The weight of plant extract per milliliter of DMSO.

### 3.3 Bacterial load

**Figure 2:**
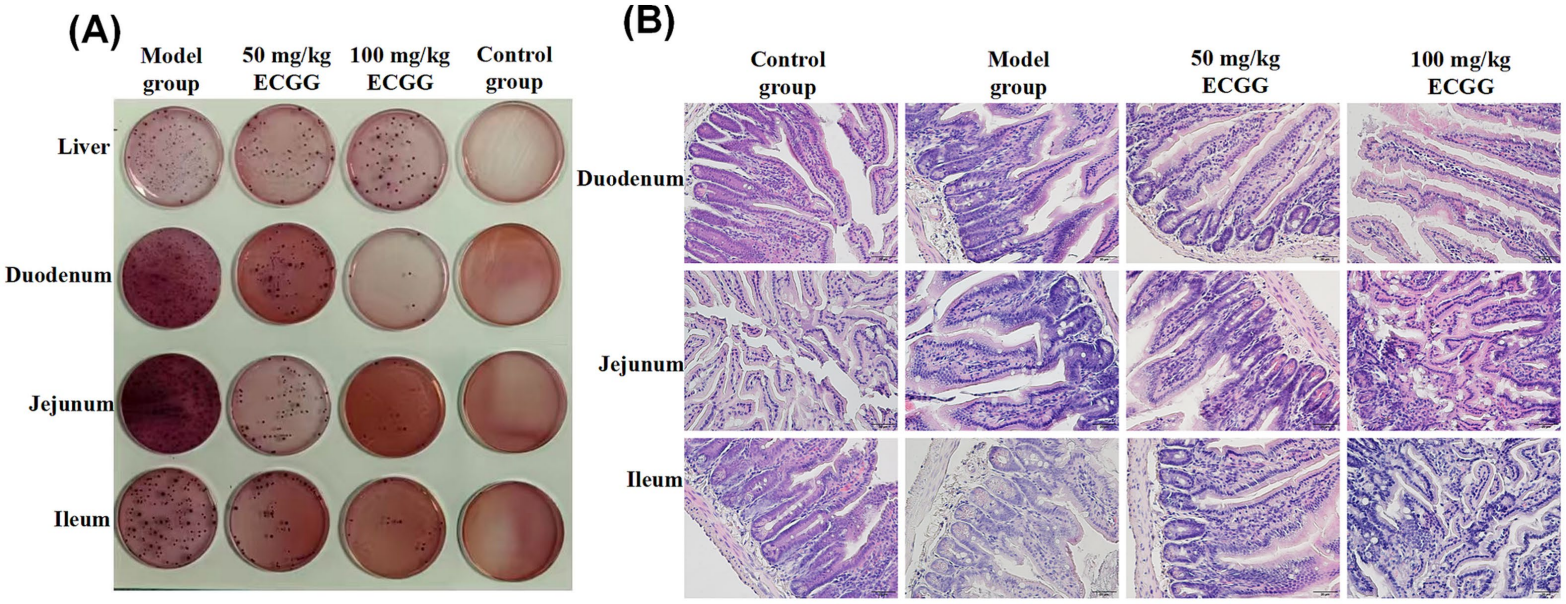
Bacterial load in mouse tissues and pathological changes of the small intestine. **(A)** Bacterial load (CFU/mL) in the liver, duodenum, jejunum, and ileum of mice. **(B)** Hematoxylin–eosin (HE) staining of mouse small intestinal tissues. All the experiments were divided into the following four groups: the control group, *E. coli*-challenged model group, 50 mg/kg EGCG treatment group, and 100 mg/kg EGCG treatment group. Scale bar = 20 μm.

After intraperitoneal injection of the fresh *Escherichia coli* culture, all mice in the model group exhibited various degrees of symptoms within a few hours, including messy fur, poor spirits, anorexia, diarrhea, trembling, chirping, pain, and huddling motionlessness. The above symptoms were alleviated in the 50 mg/kg and 100 mg/kg EGCG treatment groups, while no obvious abnormal symptoms were observed in the control group. The changes in bacterial load (CFU/mL) in homogenized tissues of mice in each group are shown in Figure 2A. The results indicated that the bacterial load in liver tissues of the model group, 50 mg/kg EGCG treatment group, 100 mg/kg EGCG treatment group, and control group was 16.7 × 10^8^, 11.1 × 10^8^, 5.5 × 10^8^, and 0, respectively; the bacterial load in duodenal tissues was 39 × 10^8^, 6.7 × 10^8^, 2.1 × 10^8^, and 0, respectively; the bacterial load in jejunal tissues was 20.6 × 10^8^, 8.3 × 10^8^, 1.6 × 10^8^, and 0, respectively; and the bacterial load in ileal tissues was 11.7 × 10^8^, 5.9 × 10^8^, 1.1 × 10^8^, and 0, respectively. The 50 mg/kg and 100 mg/kg EGCG treatment groups significantly reduced the number of bacterial colonies in the liver, duodenum, jejunum, and ileum tissues of mice. In other words, EGCG treatment significantly decreased the bacterial load in the liver, duodenum, jejunum, and ileum of *E. coli*-infected mice, indicating that EGCG can alleviate the bacterial load in the tissues of *E. coli*-infected mice to a certain extent.

### 3.4 Effect of EGCG on pathological changes in the small intestine of *Escherichia coli*-infected mice

Gross observation revealed that the small intestine of mice in the model group showed mild hyperemia, edema, thickened intestinal wall, and increased mucus, while the lesions were alleviated after drug administration, and no lesions were observed in the control group. The results of HE staining (Figure 2B) showed that the intestinal tissue of the control group had a normal morphological structure, with neatly arranged intestinal villi and intact epithelial cells. In the model group, the intestinal villi were significantly atrophied, shortened, and exfoliated, with degeneration and necrosis of epithelial cells, and inflammatory cell infiltration was observed in the lamina propria. Compared with the model group, the degree of intestinal villi damage in the 50 mg/kg and 100 mg/kg EGCG treatment groups was significantly reduced, with fewer epithelial cell lesions and less inflammatory cell infiltration. Among them, the 100 mg/kg EGCG treatment exerted the most significant effect in alleviating the damage.

### 3.5 Expression of tight junction proteins Occludin, Claudin-1, and ZO-1 in the mice small intestine

**Figure 3:**
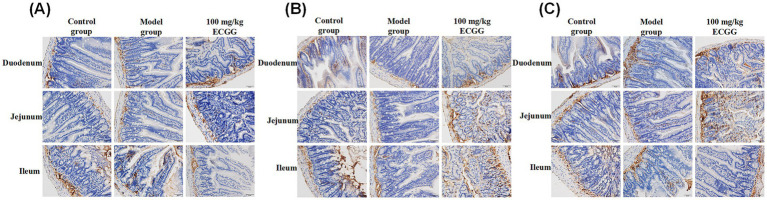
Immunohistochemical localization of tight junction proteins (Occludin, Claudin-1, ZO-1) in the mouse small intestine. Immunohistochemical staining results of the occludin **(A)**, Claudin-1 **(B)**, and ZO-1 **(C)**. Positive staining appears as brown-yellow. Scale bar = 20 μm.

As shown in Figure 3A, Occludin protein was mainly distributed in the submucosa, with a small amount in the lamina propria of intestinal villi and the muscular layer, across different groups in the duodenum, jejunum, and ileum. As depicted in Figure 3B, the distribution pattern of Claudin-1 protein in the duodenum, jejunum, and ileum among different groups was basically similar to that of Occludin protein. However, Claudin-1 protein was distributed to varying degrees in the intestinal villus mucosal epithelium of each small intestinal segment across different treatment groups. Among them, more Claudin-1 protein was observed in the villous mucosal epithelium in the 100 mg/kg EGCG treatment group, while the distribution of Claudin-1 protein was the least in the model group. As illustrated in Figure 3C, the distribution of ZO-1 protein in the duodenum, jejunum, and ileum among different treatment groups was similar to that of Claudin-1 protein.

### 3.6 Effect of EGCG on the expression of Occludin, Claudin-1, and ZO-1 proteins in the small intestine of *Escherichia coli*-infected mice

**Figure 4:**
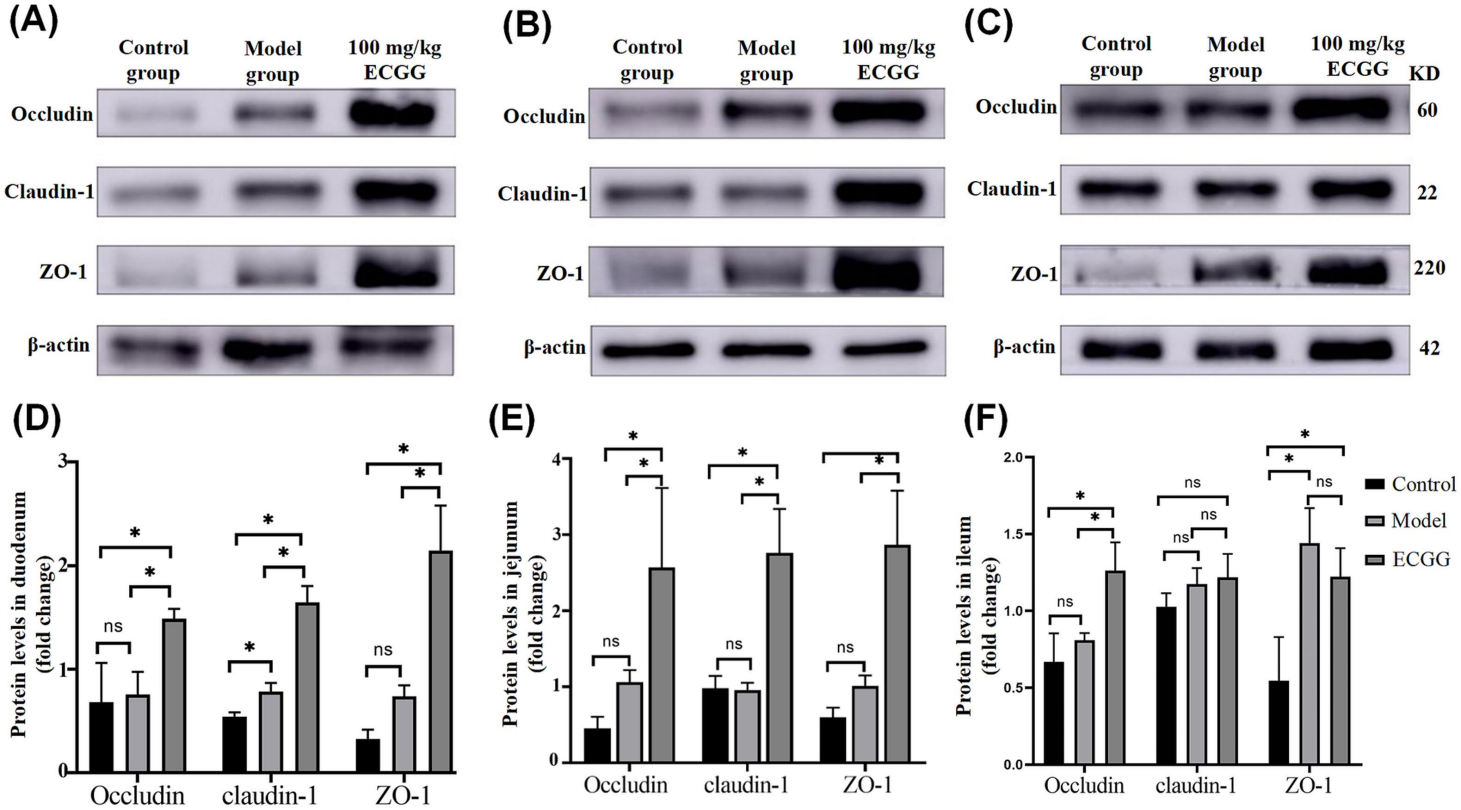
Expression levels of tight junction proteins (Occludin, Claudin-1, ZO-1) in the mouse small intestine detected by Western blotting. **(A–C)** Representative Western blot images of Occludin (60 kDa), Claudin-1 (22 kDa), ZO-1 (220 kDa), and internal reference *β*-actin (42 kDa) in the duodenum **(A)**, jejunum **(B)**, and ileum **(C)**. **(D–F)** Quantitative analysis of relative protein expression in the duodenum **(D)**, jejunum **(E)**, and ileum **(F)**. ns = no significant difference (*p >* 0.05); *p < 0.05. All statistical comparisons were performed using one-way ANOVA with Tukey’s post-hoc test.

As shown in Figures 4A,D, compared with the control group, there was no significant difference in the expression of Occludin protein in the duodenum of mice in the model group (*p >* 0.05). However, the expression level of Occludin protein in the duodenum of mice in the 100 mg/kg EGCG treatment group was significantly increased (*p <* 0.05), and meanwhile, the expression level of Occludin protein in the duodenum of mice in the 100 mg/kg EGCG treatment group was significantly higher than that in the model group (*p <* 0.05). The expression of Claudin-1 protein in the duodenum of mice in different groups showed significant differences (*p <* 0.05): the expression level in the model group was higher than that in the control group, and the expression level in the 100 mg/kg EGCG treatment group was higher than those in both the model group and the control group. The expression changes of ZO-1 protein in the duodenum of mice in different groups were similar to those of Occludin protein: there was no significant difference between the control group and the model group, while the expression level in the 100 mg/kg EGCG treatment group was significantly higher than those in both the model group and the control group (*p <* 0.05). The expression changes of Occludin, Claudin-1, and ZO-1 proteins in the jejunum of mice in different groups were similar. As shown in Figures 4B,E, there was no significant difference between the control group and the model group (*p >* 0.05), while the expression level in the 100 mg/kg EGCG treatment group was significantly higher than those in both the model group and the control group (*p <* 0.05). As shown in Figures 4C,F, the expression of Occludin protein in the ileum of mice in different groups was similar to that in the duodenum and jejunum. There was no significant difference in the expression of Claudin-1 protein in the ileum of mice in different groups (*p >* 0.05). Compared with the control group, the expression levels of ZO-1 protein in the ileum of mice in both the model group and the 100 mg/kg EGCG treatment group were significantly increased (*p <* 0.05), while there was no significant difference in the expression of ZO-1 protein in the ileum between the model group and the 100 mg/kg EGCG treatment group (*p >* 0.05).

## 4 Discussion

Calf diarrhea is a prevalent disease in livestock production, and *Escherichia coli* (*E. coli*)—a primary pathogen responsible for diarrhea in young livestock (2, 3)—is a Gram-negative, rod-shaped bacterium that forms red colonies on MacConkey agar. Its prevalence and diversity in livestock have been documented in recent epidemiological studies (4–6). Its major pathogenic types include Enterotoxigenic *E. coli* (ETEC) and Enteropathogenic *E. coli* (EPEC), and co-transfer of resistance genes such as mcr has been reported in livestock isolates (13). Severe infections can directly cause livestock death, impair dairy and meat production, and result in substantial economic losses. Studies have shown that *E. coli* infection induces clinical symptoms such as anorexia, lethargy, dehydration, and watery feces, leading to reduced growth rates and compromised production performance in animals (14–16). In this experiment, the clinical symptoms of mice challenged with *E. coli* were highly consistent with these findings, verifying the scientificity and reliability of the constructed experimental model. This study successfully simulated the pathological process of natural *E. coli* infection, laying a foundation for subsequent investigations into the protective mechanisms of plant extracts against *E. coli*-induced intestinal damage.

Plant extracts, valued for their natural safety and multifunctionality, exert anti-*E. coli* effects via inhibiting enterotoxin synthesis and creating a hostile microenvironment for pathogens (17, 18). Their bactericidal activity involves multi-target actions, including disrupting cell wall/membrane integrity, interfering with protein function, damaging mitochondria, and blocking ATP synthesis (19). Our data confirmed significant *E. coli* growth inhibition by EGCG, shikonin, and resveratrol—consistent with Cuimin et al. (20), who reported enhanced EGCG bactericidal activity under high Ca^2+^ conditions, supporting the universality of plant extracts’ multi-target mechanisms. While Megan et al. (21) and Xiang et al. (22) also demonstrated shikonin/resveratrol’s efficacy, MIC discrepancies existed (e.g., resveratrol: 3.125 mg/mL herein vs. 1 μg/mL in Xiang et al.’s study). These variations likely reflect differences in bacterial strains, extract sources, or applied concentrations (23). The use of a clinically relevant calf-derived *E. coli* isolate in this study adds strain-specific value to the existing literature.

After piglets are infected with ETEC K88, intestinal villi undergo marked atrophy, causing a severe imbalance in the villus height-to-crypt depth ratio. Meanwhile, the surface area of the jejunum and ileum is significantly reduced, damaging intestinal mucosal integrity (24). These pathological changes severely impair nutrient digestion and absorption, adversely affecting piglet growth, development, and health. Currently, antibiotics are the primary preventive and therapeutic approach, but their rapid bactericidal effect is accompanied by risks such as antimicrobial resistance (AMR) and drug residues (25).

In contrast, plant extracts inhibit inflammatory responses via multiple mechanisms—reducing inflammatory cytokine production, regulating immune function, optimizing intestinal microecology, and alleviating intestinal inflammation. These effects improve nutrient digestion and absorption, increase final body weight and average daily gain (26), making plant extracts potential alternatives to traditional antibiotics. For example, He et al. (11) and Zhou et al. (27) demonstrated that EGCG plays a key role in preventing and treating intestinal inflammation, improving intestinal barrier function, and regulating the expression of anti-inflammatory cytokines and tight junction proteins. Wang et al. (12) confirmed EGCG’s strong anti-inflammatory, antioxidant, and antibacterial activities, while Dan et al. (28) found that EGCG disrupts *E. coli* cell membranes, enhances inner and outer membrane permeability, and exerts significant inhibitory effects.

Consistent with these findings, mice in the model group exhibited typical pathological features post-infection, including intestinal tissue hyperemia and edema, thickened intestinal walls, villus atrophy and shedding, and epithelial cell degeneration and necrosis. However, EGCG treatment significantly alleviated lesion severity and reduced intestinal bacterial load. These results further verify EGCG’s efficacy in preventing and treating *E. coli* infections, suggesting that plant extracts may serve as ideal alternatives to traditional antibiotics for *E. coli*-associated diseases—providing important insights for the development of green and safe novel antibacterial agents.

The intestine is not only the primary organ for nutrient digestion and absorption but also a key natural barrier for maintaining systemic homeostasis. Pathogen invasion induces intense intestinal oxidative stress, leading to intestinal epithelial cell damage that impairs intestinal structural integrity and compromises animal health and growth performance (29). *E. coli* invasion also severely disrupts intestinal microbial homeostasis: ETEC infection reduces the abundance of beneficial flora such as Bacillus, Lactobacillus, and Enterococcus (2, 3). As a critical component of the intestinal immune barrier, the intestinal flora regulates immune function and resists pathogen invasion (30).

The intestinal barrier comprises intestinal epithelial cells and intercellular tight junction proteins, acting as a physical defense against external harmful substances and maintaining epithelial integrity (15). Tight junctions form a complex network consisting of transmembrane proteins (e.g., Claudin-1, Occludin, JAM-A), cytoplasmic proteins (e.g., ZO-1, ZO-2), and cytoskeletal proteins (31).

Adverse stimuli alter the expression and localization of tight junction proteins, disrupting tight junction structure, impairing barrier function, increasing intestinal permeability, and triggering bacterial translocation and inflammation (30). For instance, *E. coli* damages intestinal barrier function by affecting Occludin, Claudin-1, and ZO-1 expression (32, 33, 35). In this study, immunohistochemical analysis showed positive expression of tight junction proteins in all small intestinal tissues of mice, albeit with subtle distribution differences—consistent with reports that damaging factors alter tight junction protein localization. This suggests potential intestinal barrier impairment, with subtle distribution changes possibly representing an early response to damage. However, the underlying molecular mechanisms by which EGCG regulates these tight junction proteins remain to be fully elucidated (37). Future studies employing proteomic or transcriptomic approaches could uncover additional targets and pathways modulated by EGCG, providing a more comprehensive understanding of its barrier-protective effects.

While the murine model used in this study is valuable for mechanistic insight, it does not fully replicate ruminant physiology. Therefore, caution is warranted when extrapolating these findings to calves. Future studies in calf models or using ruminant intestinal organoids are necessary to validate the translational potential of EGCG. Nevertheless, the present work provides a strong foundation for further exploration of EGCG as a natural antibiotic alternative in livestock.

In terms of practical application, several challenges remain, including the bioavailability of EGCG in the gastrointestinal tract, optimal formulation for oral delivery, and regulatory approval for use in food-producing animals (38). Despite these hurdles, our results support the potential of EGCG as a candidate for controlling colibacillary diarrhea in calves, and further research addressing these translational aspects is warranted.

## 5 Conclusion

This study confirmed that epigallocatechin gallate (EGCG) exhibits significant antibacterial activity against a clinically isolated calf diarrhea-causing *Escherichia coli* causing calf diarrhea, with an inhibition zone diameter of 19.7 ± 0.3 mm and a minimum inhibitory concentration (MIC) of 3.125 ± 0.0 mg/mL. Hematoxylin–eosin (HE) staining demonstrated that EGCG could significantly alleviate intestinal pathological damage. Further analysis using immunohistochemistry and Western blotting revealed slight changes in the localization of Occludin, Claudin-1, and ZO-1 proteins. Compared with the control group and model group, EGCG significantly increased the expression levels of these three tight junction proteins, indicating that EGCG exerts a positive protective effect on small intestinal damage in *E. coli*-infected mice. These findings provide a scientific basis for further development of EGCG as a natural antibiotic alternative for controlling calf diarrhea, although additional studies are needed to address translational challenges and mechanistic details.

## Data Availability

The original contributions presented in the study are included in the article/Supplementary material, further inquiries can be directed to the corresponding author.
