# Supplementary material for: Antibacterial effects of epigallocatechin gallate against calf diarrhea-causing Escherichia coli: in vitro and in vivo investigations
Source: Front Vet Sci. 2026 Apr 9;13:1752084. doi: 10.3389/fvets.2026.1752084 (PMC13102593; doi:10.3389/fvets.2026.1752084)

**Western Blot Instructions and Original Images**

At first, we check the full films of western blot for the protein samples of rooster testicles to ensure the specificity of antibodies. The results were showed as follows:


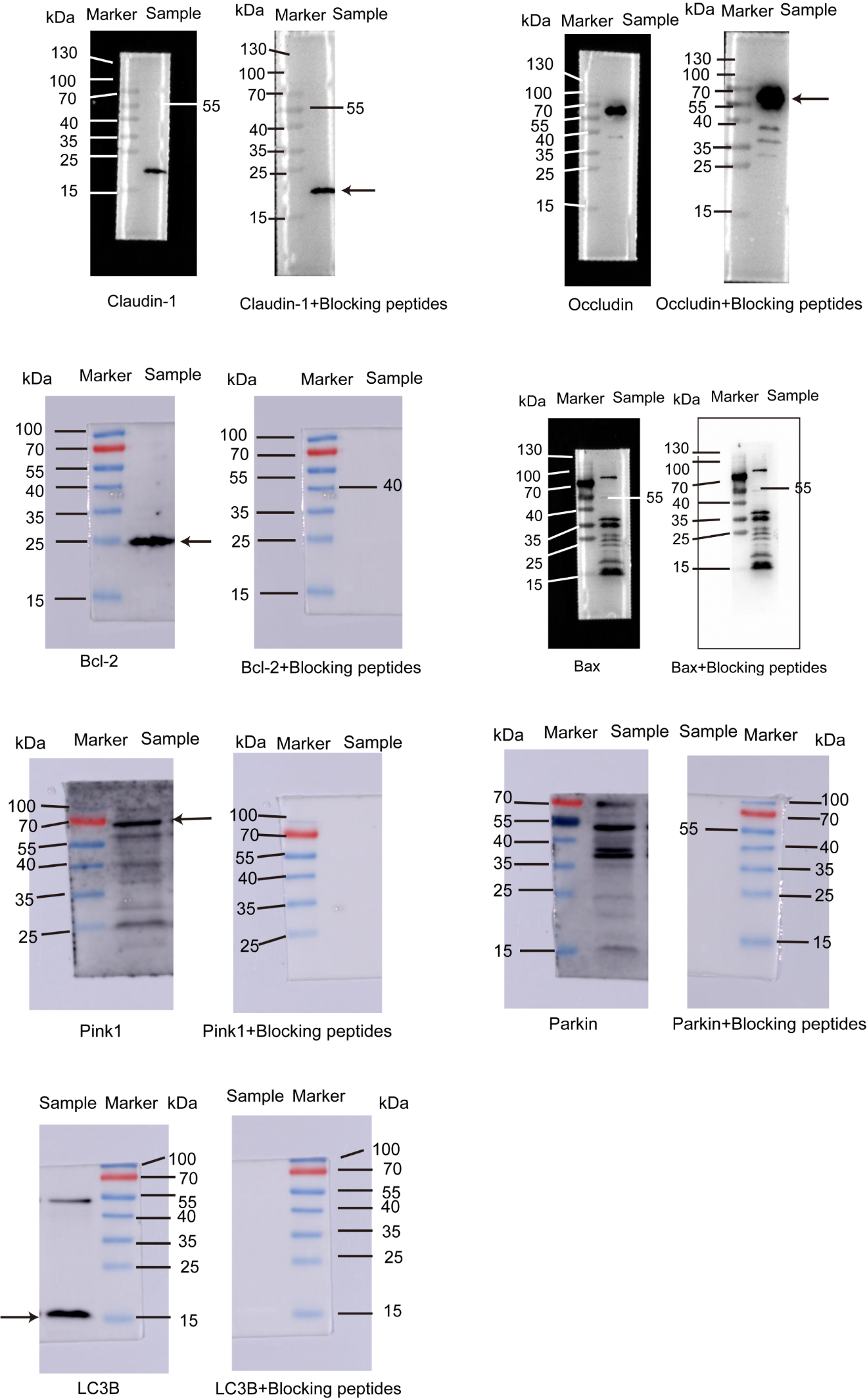


After ensuring the specificity of the anti-Claudin-1, anti-Occludin, we performed following experiments in article with cropped gels. All original images were showed as follows:

Western blot original images of the Claudin-1, Occludin and ZO-1 proteins in the testes of figure 4.

**(A) Duodenum**

Occludin 60 kDa


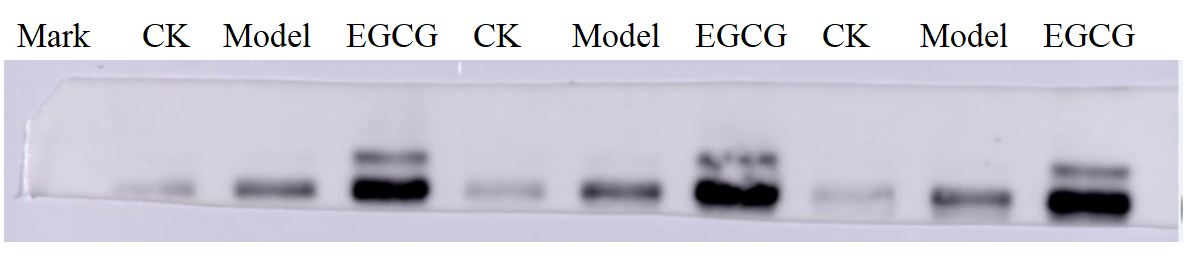


Claudin-1 22 kDa


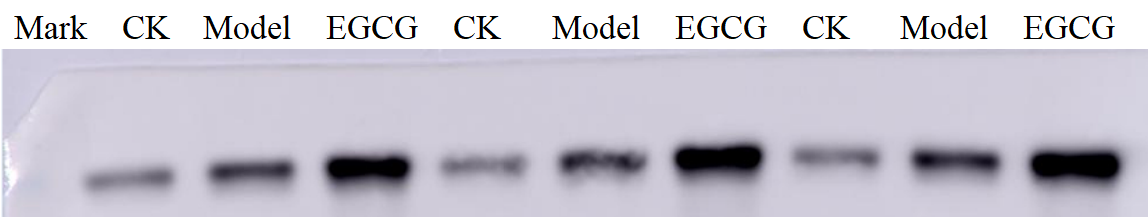


ZO-1 220 kDa


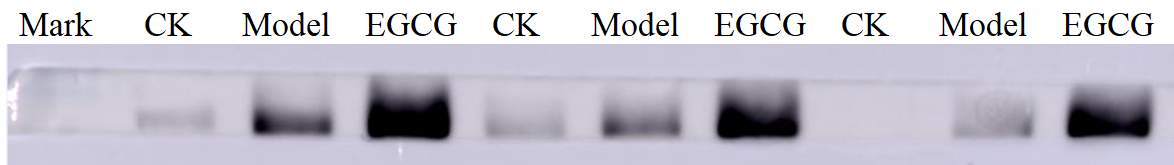


Occludin 60 kDa

β-actin 42 kDa


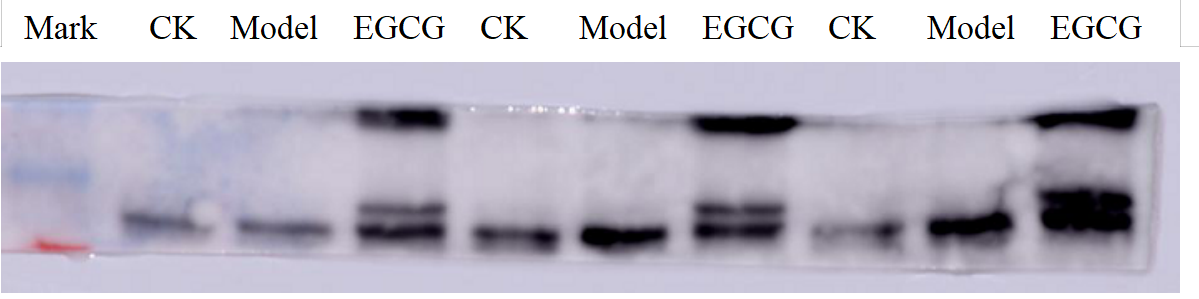


1. **Jejunum**

Occludin 60 kDa


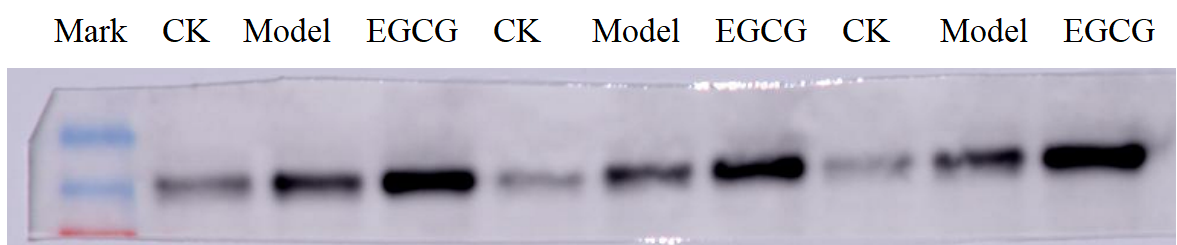


Claudin-1 22 kDa


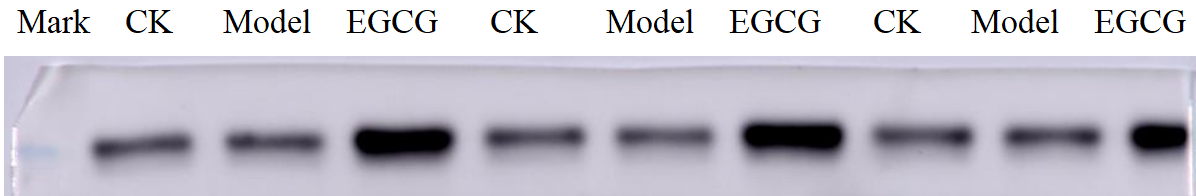


ZO-1 220 kDa


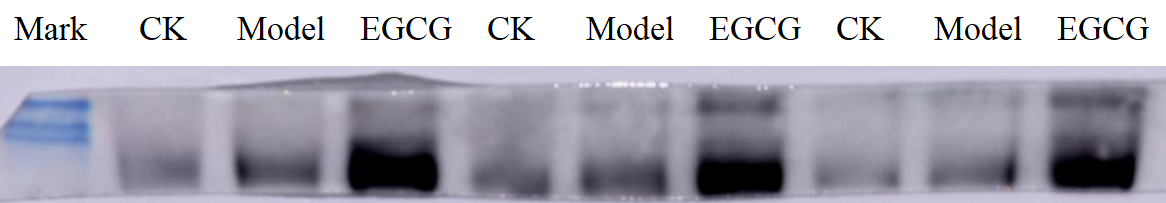


β-actin 42 kDa


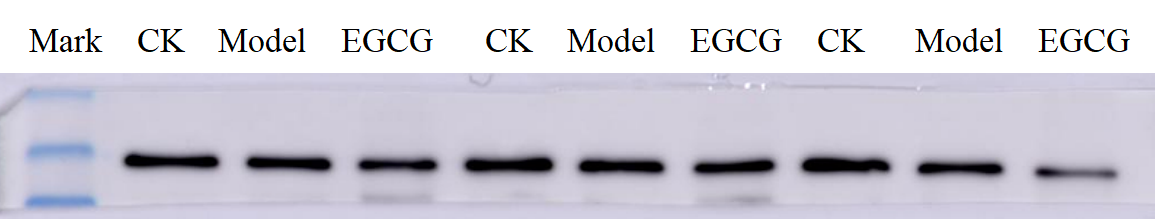


**(C)Ileum**

Occludin 60 kDa


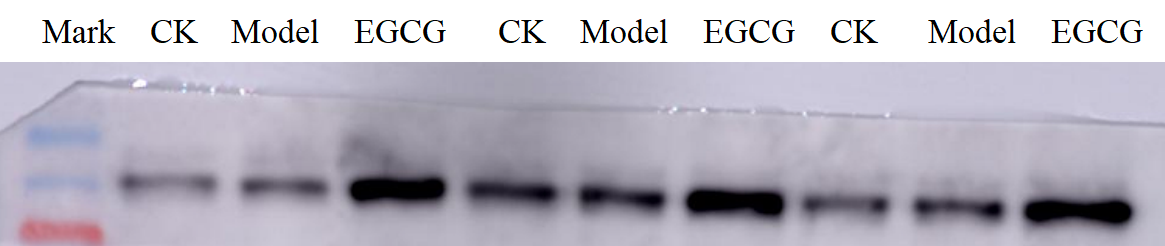


Claudin-1 22 kDa


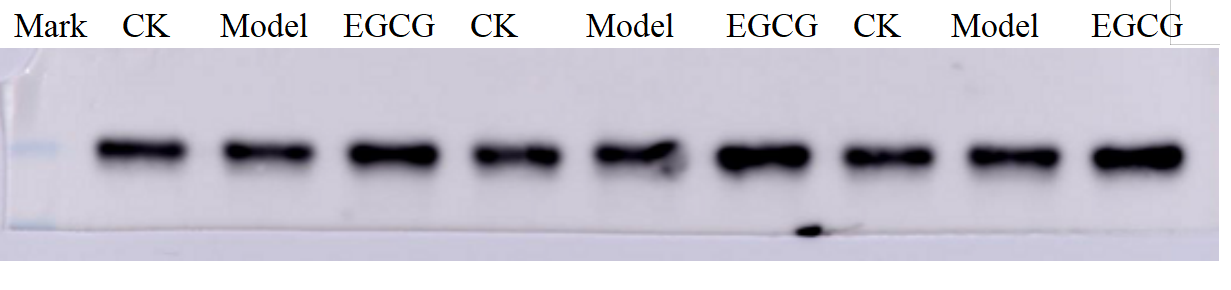


ZO-1 220 kDa


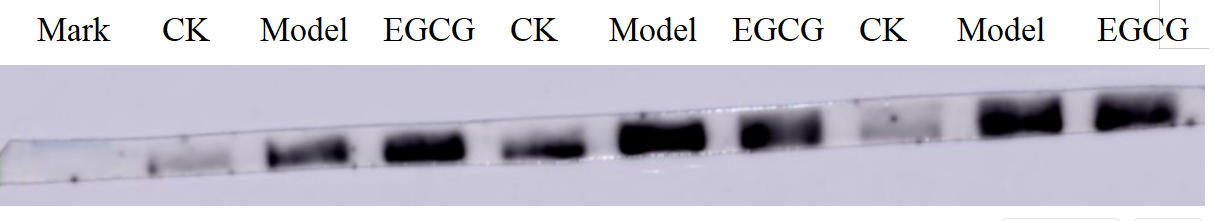


β-actin 42 kDa


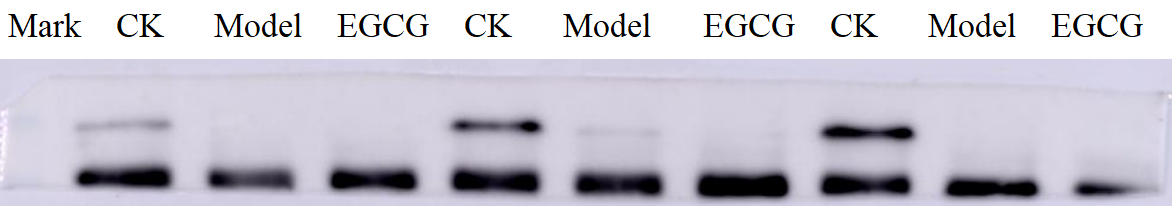

Supplement: Supplementary file 1 [file Data_Sheet_1.DOC]
